# Patient Perspectives on Inpatient Mealtimes: Insights on Swallowing, Mental Wellbeing and Recovery

**DOI:** 10.1111/inm.70212

**Published:** 2026-01-05

**Authors:** Susan Guthrie, John Baker, Jane Cahill, Bronwyn Hemsley

**Affiliations:** ^1^ Leeds and York Partnership NHS Trust Leeds UK; ^2^ School of Healthcare University of Leeds Leeds UK; ^3^ Graduate School of Health, Faculty of Health University of Technology Sydney (UTS) Ultimo Australia

**Keywords:** dysphagia, eating, mental disorder, patient‐centred care, psychiatry

## Abstract

Although the prevalence of swallowing difficulties in adults with mental health conditions has been highlighted in research, the relationship between swallowing difficulties and psychosocial aspects of mealtime experiences is not known. In the context of a need for person‐centred and holistic approaches to nursing and healthcare, the paucity of research relating to the impact of mealtimes on hospital wards on patient wellbeing and safety reveals a significant gap in knowledge. To understand patient views and experiences of mealtimes on inpatient mental health wards, including both positive and negative aspects affecting their wellbeing and safety, this ethically‐approved study was qualitative in design and involved collaboration with service‐user groups who co‐designed interview questions. Participants were 13 inpatients on mental health wards for working‐age adults. Interviews were conducted in person, de‐identified and analysed using thematic analysis. Themes were first constructed from transcripts and then verified with service‐user groups and the research team. The overarching theme of ‘heightened emotions associated with mealtimes’ encapsulated four themes: ‘experiencing anxiety and stress, connecting with others at mealtimes, loss of autonomy and choice, and experiences of swallowing difficulty’. Heightened emotions, anxiety and stress experienced during mental health ward mealtimes were counter to patients' wellbeing, which impacted on their wider recovery. Mealtime and swallowing safety, particularly when dysphagia is present, may be negatively affected by emotional pressures during mealtimes. These findings suggest a need to address barriers to wellbeing at mealtimes, and to raise awareness around integrating mealtimes into recovery pathways and person‐centred care.

## 1 Background

Choking and swallowing difficulties are known to be prevalent in populations of adults with mental illness (Aldridge and Taylor 2012; Chen et al. 2015; Cicala et al. 2019). The impact of swallowing difficulties on individual patients with mental illness on hospital wards is poorly understood, as is how to provide appropriate personalised support at mealtimes. Mealtimes on inpatient mental health wards impose different environments and social constraints compared to non‐hospital experiences.

Prior to hospital admission, and continuing during the duration of stay, each person's cultural, spiritual and personal customs at mealtimes are powerful influences on their psychosocial wellbeing. The role of food and drink, and the activities of eating and drinking in adult life are both individual to the person and common across communities, contributing to mental as well as physical wellbeing (Shelley 2020). However, on admission into a hospital mental health ward, the individual is faced with new mealtime routines which include different and potentially challenging experiences, unpredictable encounters with other patients, and restrictions to mealtime choices and procedures. Unlike acute NHS physical health wards, mentally ill patients have significantly longer hospital stays than other patients (Gilburt and Mallorie 2024). In hospital, these patients are usually expected to sit at tables for meals, in a shared dining room, receiving food from staff with varying levels of engagement. Menu options are chosen in advance (sometimes days ahead), and restrictions may be in place for safety and security (e.g., increased staff supervision, wipe‐clean furniture secured in place, removal of glass or metal objects).

Mental health ward patients' experiences of mealtimes may be further compromised by choking and dysphagia (swallowing difficulties), which are highly prevalent in this group (Aldridge and Taylor 2012; Regan et al. 2006). Physical aspects of eating, drinking and swallowing difficulties have been discussed relating to adults with mental illness (Bazemore et al. 1991; Kulkarni et al. 2017; McManus 2001). The emotional impact of such difficulties on these patients is poorly understood, as current research on swallowing in this population has focussed almost exclusively on iatrogenic and neurological factors (Guthrie et al. 2023). Research considering psycho‐social experiences of mealtimes on mental health wards is scant (Guthrie et al. 2023).

In the UK, incidents of choking in inpatient settings must be reported documenting the clinician's immediate observations (NHS England 2024). However, the wider context and factors leading up to choking incidents are rarely described (Guthrie 2025; Hemsley et al. 2019). In addition, patients' perspectives describing the impact of swallowing difficulties and/or choking on their quality of life and mental wellbeing are typically not included in incident reporting systems (Guthrie et al. 2015). Understanding patient experience, supporting inclusive approaches and shared decision making has been discussed in relation to good practice in supporting people with mental illness working towards recovery (National Institute for Health and Care Excellence 2021). However, such collaborative approaches are not apparent in current literature describing mealtimes, dysphagia assessment and dysphagia intervention for adults with mental illness.

## 2 Aims of the Study

The aims of this collaborative research were to understand patient perspectives on mealtime experiences and swallowing difficulties on mental health wards, including consideration of both positive and negative aspects that may influence inpatient experiences and wellbeing.

## 3 Methods—Study Design

Ethical approval was provided by the Health Research Authority and local sites of investigation (IRAS project ID number 270116) and the supervising University.

The qualitative study was co‐designed in consultation with patients and caregivers. This involved the first author meeting with two patient and caregiver advisory groups to discuss the aims and methods including design of interview protocols. The group members prioritised topic areas for investigation, considered options for data collection and co‐designed questions for in‐depth interviews with adults on mental health wards (see Table 1). Terminology was discussed and the consensus from group members was to use language reflecting the participants' hospital environment (e.g., ‘mental illness’, ‘patient’). This pragmatic exploratory study followed Thorne's interpretive description approach highlighting the importance of individualised experiences (Thorne 2016). The participants were a marginalised and vulnerable group. Current NHS guidance and reports identify people with mental illness as a priority area and as people who are facing health inequality, particularly in accessing timely and continuity of care (Department of Health and Social Care 2025; Mason et al. 2025; Royal College of Nursing 2025). This study supported patients to voice their concerns and insights addressing the concept of ‘silences’ proposed by Serrant‐Green (2011) as a theoretical basis to explore the needs of marginalised populations.

**TABLE 1:** Interview questions and prompts.

Warm up item for discussion: ‘please could you describe your favourite meal?’	
(1) Can you tell me what it's like on this ward at mealtimes?	Prompts: to expand on setting, staff activity, atmosphere at mealtimes, social aspects
(2) How does this compare to having a meal at home?	Prompts: to expand on personal ‘normal’ compared to ward meals, own cultures and behaviours
(3) Have you ever worried about personal safety at mealtimes? Have you seen anyone have difficulty?	Prompts: to expand on any concerns or anxieties, any comments on how to improve the mealtime experience, any signs of swallowing difficulty
Offer images to support each question showing diversity in settings, ages and ethnicity.	

### 3.1 Participants

Eligible participants were working age adults (aged 18 to 65 years), capable of giving informed consent and recruited from inpatient mental health wards from one city in northern England. Only participants with a primary diagnosis of mental illness were eligible. People with a diagnosis of eating disorders were excluded due to their need for additional specialist support around eating and drinking. People over the age of 65 were not eligible for this study; the focus was centred on the insights, needs and cultures of working age adults.

Inpatients with mental health conditions (13 in total), aged between 20 and 60 years, took part in this study on a range of acute, forensic and rehabilitation wards (including locked and open access hospital accommodation). All participants were deemed capable of giving informed consent and able to communicate verbally at interview. Capacity to consent was determined by ward staff familiar with each participant's current presentation. This ensured the person's acuity of mental illness, ability to engage in interview and general wellbeing were considered and supported. The eight males and five females were majority white British; other ethnic groups and cultures included African, Asian, Caribbean and Gypsy. Eight of the participants reported active religious beliefs and practices. No other background data is reported in order to protect privacy and confidentiality. Reference codes for each participant are given following each quote, in the format ‘Patient number’.

### 3.2 Data Collection

The individual interviews were conducted in person between May 2021 and July 2022. Duration varied from 6.04 to 41.58 min (total 251.29 min). The interview questions were modified each time to adapt to individual participants' communication and intellectual capabilities. The questions were open to allow patients to offer their insights for all aspects of the mealtime experience. Their responses reflected their priorities and main areas of concern (Table 1).

### 3.3 Ethics

The researchers were mindful of the vulnerability of the participants with mental illness living under Section (Mental Health Act 1983) on the wards. Patient information was drafted in discussion with the service‐user groups to promote accessibility and understanding. The researcher observed for signs of distress or agitation during the interview and it was made clear to each participant that they were able to withdraw from the study at any stage without any impact on their treatment and care. Interview data in the form of audio recordings and transcripts were saved into secure storage compliant with university and NHS protocols for the period of the study then deleted. Anonymised transcripts were stored, with participant consent, in the University data repository with restricted access for up to 10 years available on request from corresponding author.

### 3.4 Analysis

Reflexive thematic analysis was used incorporating the first author's viewpoint as a clinician and following a pragmatic approach (Braun and Clarke 2021). Reflexivity underpinned this analysis, acknowledging the position of the lead author as an ‘insider’, working as an experienced clinician on the wards where the research was conducted. The first author conducted the interviews, transcribed each from audio recordings and completed inductive coding of the participant narratives in consultation with the research team who are co‐authors. NVIVO was used to store and retrieve data facilitating iterative analysis of the participants' insights, identification and definition of codes then connecting these into themes. To address trustworthiness the COREQ checklist was used (Tong et al. 2007): mapping the study against the COREQ domains (see Appendix S1) and evaluated with reference to quality guidance around use of reflexive thematic analysis (Braun and Clarke 2023). This promoted rigour of process, clarification of context and transparency.

## 4 Results

### 4.1 Overarching Theme: Heightened Emotions

Participants' interview transcripts revealed strong emotions associated with mealtimes relating both to their time on the ward and prior to admission. The intensity of emotion was apparent in the emphasis and content of the descriptions of home vs. ward as places to eat, the reactions to the presence of others, attitudes to the mealtime experience, and experiences of swallowing difficulties. The descriptions of inpatient mealtimes were largely negative, reporting raised levels of stress and anxiety. In stark contrast, reports of home mealtimes focussed on more positive aspects, including flexibility, choice and a source of comfort with sharing food and/or drink to build and maintain relationships. Patients described the emotional benefits of home mealtimes:

… its comfort food you know, soothing food …. Soothing food, tastes nice, and gives you a warm feeling … Yeh, gives you a warm feeling when you eat it … it's what you want if it's the right type of food. (Patient 02)

**FIGURE 1:**
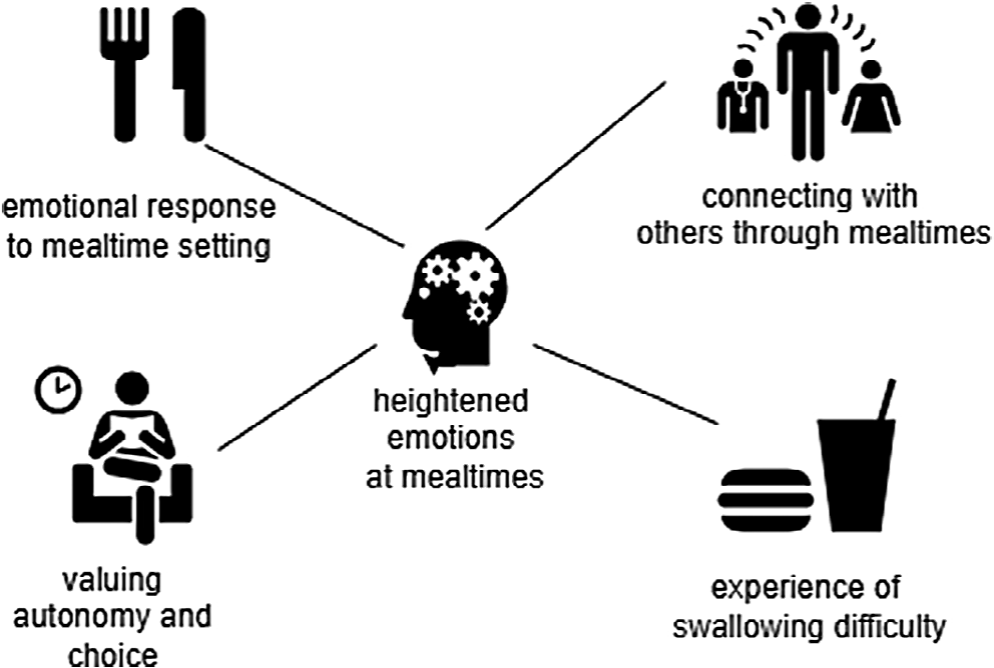
Thematic network of findings.

Within the overall theme of ‘heightened emotions at mealtimes’ there were four main themes shown in Figure 1.

### 4.2 Theme 1 Emotional Responses to the Mealtime Setting

The first comments offered in the interview typically concerned frustrations and tensions associated with the ward mealtimes. Spontaneous comments gave a sense of generally unpleasant mealtime experiences: ‘It's stressful and it shouldn't be stressful’ (Patient 12). The negative emotions often related to specific aspects of the dining room relating to both the people present and the room. Lack of space was a primary concern due to seating around shared tables. Crowded dining rooms resulted in close contact between patients during mealtimes and a lack of choice in seating. For those patients preferring more space or quieter environments, this became intensely stressful. In describing ward mealtime environments, participants used vocabulary such as ‘busy’ (Patient 01), ‘crush’ (Patient 09), ‘hectic’ (Patient 13), prompting emotional responses such as ‘stressful’ (Patient 12), ‘uncomfortable’ (Patient 09), and ‘nervous’ (Patients 06 and 11). Their accounts revealed frustration and some reported avoiding the meal altogether.

The queuing system at mealtimes presented additional emotional challenges, with increased frustration if not receiving the correct food as ordered. Participants' descriptions conveyed a sense of pressure to keep a place in line close to other patients. Anxiety was also reported for those at the back of the queue who reported worrying about missing out on meal options:

Queuing up … and it's like … first come first served …Yeh so everyone's rushing in to … queue and it's like … you would get what's left over. (Patient 03)

A further source of stress was the unpredictable and unappealing nature of the food itself. For many, the food was repetitive and lacking in variety, reflecting the institutional nature of the setting. Some participants described choosing to eat in their bedrooms in preference to coming into the dining room. For some this resulted in most ward meals being replaced with takeaway meals or snacks.

### 4.3 Theme 2 Connecting With Others Through Mealtimes

Many participants highlighted the important role of mealtimes in facilitating connections with others. This included building and maintaining relationships with family and friends and, when in hospital, potentially with other patients and staff. However, most participants described preferences for eating alone on the ward and to avoid interacting with others. Some who had avoided other patients outside of mealtimes were faced with the need to tolerate them and staff for the duration of the meal.

Some participants described showing care for others by offering food or drink as an important part of maintaining relationships and personal wellbeing prior to their admission to the ward. They valued these previous experiences of cooking and caring for family or friends, which contrasted starkly with the sense of isolation for mealtimes on the ward:

You go into the kitchen, or the dining room and you put the kettle on, and you just make a cup of tea [Researcher asks: “does that kind of bring people together do you find … in there?”] No, no … it's solo experience. (Patient 02)

Family mealtimes at home were described with mixed emotions. For the most part, patients described family mealtimes affectionately: as a time for family to be together and a highlight of the day. This compared negatively to inpatient mealtimes:

For me … it's never been the same since I've not cooked for me children. (Patient 12)

When describing home mealtimes, there was an emphasis on connections and relationships and validating mealtime cultures and customs. This is discussed further below exploring the importance of personal cultures associated with mealtimes (Theme 3).

Occasionally, emotional aspects of mealtimes were commented upon negatively in relation to earlier home life and childhood experiences. For one participant this included breakdown of relationships:

They [mealtimes] can be awkward, weird family, … even though … broken families and stuff. (Patient 01)

Despite the comments above acknowledging that mealtimes have the potential to build connections and support relationships, most participants in this study did not relate any aspect of ward mealtimes to improving their current relationships. Nor did they consider that mealtimes on the ward could contribute to recovery or progress in mental wellbeing. Interactions with other patients were scant, with few reports of social exchanges with staff during mealtimes. Rather, participants described difficulties connecting with staff who were sitting at separate tables, busy supervising menu choices for the next day, and organising queuing systems. This was interpreted by some participants as being related to the pressures of work, and by others as staff actively choosing not to interact. Participants perceived the staff role to be more about monitoring adherence to safety protocols, behaviour management and maintaining orderly mealtime routines:

Yeh because [catering] want to wash up and they want to go, like… when alarm goes off, yeh … the staff have to run off the ward and go on a different ward. [Researcher asks: “so what happens if you're in the middle of your dinner?”] “Er, we have to leave it … Not nice.” (Patient 08)

Patient 08 emphasised the organisational pressures overriding individual concerns and choices affecting both staff and patients. Pressures experienced by patients on the wards were also derived from close proximity to other patients triggering negative emotions including stress, nervousness, disgust, anxiety and/or fear. Being near to someone with deteriorating mental illness was difficult:

… the only available place [in the dining room] was with somebody who wants to fight all the time and uses F words all the time, so I thought I'll go and try eating in my room. (Patient 07)

Other patients' habits at the dining tables were also described as unpleasant and difficult to tolerate. Some participants described feeling self‐conscious about their own behaviours at mealtimes and feeling embarrassed to be with others at that time. Others described their wider anxiety relating to deteriorations in their own mental health:

I just, I get right nervous in front of people's company … I just get really really clammy hands and that … don't like meeting lots of people. (Patient 06)

The close dining room environment was perceived to exacerbate relationship issues between patients. Prior negative interactions and social difficulties could not be avoided when the meal was on offer in one room only:

… with that woman, I was just like treading on eggshells … there was one time when, like, I sat near her and stuff and then it, like, it escalated into, like, an argument and stuff like that. (Patient 13)

The concerns related most often to uneasiness around other patients but in some cases, participants voiced concerns around staff who were watching and supervising:

(Researcher asks, “what do you think the staff are watching for?”) “I don't know … to make sure nothing gets out of hand, or nobody can be themselves or something … they don't like it if you be yourself.” (Patient 05)

This account highlighted the differences between hospital and home mealtimes and captured the sense of negativity and loss of identity. This is explored further in the theme below.

### 4.4 Theme 3 Valuing Choice and Autonomy at Meals

This theme related to patients' insights on control around choices for food and drink and mealtime customs. Personal traditions and beliefs were keenly valued and described as important for mental wellbeing. Ward restrictions and routines were acknowledged as necessary but remained a source of frustration when conflicting with individual preferences and customs. Participants reflected on how cooking their own food brought emotional benefits and certainty as well as improved tastes:

I prefer me cook my meal myself … Cos I just cook, and I know it's ok. (Patient 09)

However, access to self‐catering was limited and permissions varied according to the person's mental wellbeing. Having to seek permission for accessing food or drink, asking for kitchens and utensils to be unlocked, and requesting staff presence to supervise kitchen access, gave a sense of dependency:

You don't want to be disturbing them … and asking them ‘can you make me a cup of tea’, like a five‐year‐old child. (Patient 02).

Participants reflected that, as adults, they would normally be able to choose time, place and food to eat. At home they described making last‐minute decisions or changing their minds about mealtimes. Many described how, in hospital, this autonomy and flexibility were lost. Being able to cook was valued in terms of achieving choice and control:

Me have more space and time [sic] … more space and time … Yeh … that's important … When you're cooking … you can sit down, you can do … you can turn off the stove, go back or do what you want to do … you have more space and time. (Patient 09)

The repetition of ‘space and time’ emphasised the participant's desire for control over the mealtime as an event. His account focused on his independence and autonomy at mealtimes, valuing mealtimes as more than just access to food and nutrition.

Participants commented on difficulties in maintaining personal mealtime customs and routines as an inpatient, suggesting that this loss of personal cultures and identity had impacted on their wellbeing in hospital. Many described mealtime traditions from home and childhood that were still important to them. Participants reported that ward catering services did acknowledge religious and other dietary needs in menus; however, selections were unpredictable and uncertain in practice. Participants perceived that staff support and attention were focused more towards the group as a whole rather than individuals:

[Researcher asks: “Do you think they take account of what individual people might want?”] “No, they just bring whatever they think is good for the patients … maybe I'm right or wrong.” (Patient 01)

Patient 07 referred to a staff member as a ‘despot’ and another suggested that staff had a ‘prison mentality’ (Patient 13) which added to the sense of patients' powerlessness on the ward. Some participants conveyed an air of hopelessness:

Er … staff are not really good; they don't really help. …. but the staff they don't really talk … they don't really talk to patients … no idea…it's sad. (Patient 01)

There was disappointment and a sense of despondency as patients faced the dining room and the meal:

Well, it makes me feel “shall I bother?” … it really does … but if you're hungry you've got to eat, but it does put me off. (Patient 12)

Participants reflected on how the setting for the meal affected their wider experience on the ward, describing the wards as detached from previous and future lives. One participant summed up mental health wards as being ‘places where you can't live your life’ (Patient 01).

### 4.5 Theme 4 Experience of Swallowing Difficulty

For most participants, the theme of heightened emotions was closely linked to experiences of swallowing difficulty. The stress and anxiety described above were exacerbated for participants who had additional concerns relating to choking and dysphagia:

The only thing that's bothering me is that choking thing, I feel like I'm going to … if I choke then I'm not going to be able to control it. (Patient 06)

Participants had observed others having swallowing difficulty but some described their own experiences of choking showing insight into the potential consequences. However, the level of concern related to choking varied. While some participants described choking experience calmly, others conveyed distress and lasting concern. One participant gave a full description of his experience of choking:

I didn't chew it properly, swallowed it … and it got stuck right across my throat … and I couldn't … (gestures to neck and mimes choking) … horrible, you think you're gonna die, it were down there about a minute and a half … now I were gasping. (Patient 02)

Participants varied in their self‐awareness of swallowing difficulties. Some could locate difficulties in swallowing identifying ‘back of the throat’ and ‘gagging’ (Patient 03). Others focussed on the difficulties in feeding themselves, for example: ‘shaking with my hands, I can't use my cutlery properly’ (Patient 10). Concerns about posture were also mentioned with some reporting difficulties in sitting upright, or in leaning forwards, and needing support for positioning. Others reported feelings of physical discomfort, or being unused to eating at a table due to previous lifestyle or culture.

Participants often described how the environmental pressures of the mealtime setting (described above) would lead to increased speed of eating. They described this as intended to reduce the time spent in an unpleasant mealtime situation and resulting from anxiety (e.g., wanting to leave a crowded dining room). However, some participants also felt that pressure to eat fast came from wider institutional tensions and staff pressures to hurry the meal so that staff could respond to alarms and other patient needs.

Participants reflected on how their mental illness affected their mealtimes, both emotionally and in relation to their physical skills in swallowing food and drink. Some identified issues relating to specific oral skills:

I was having a problem with drooling … it felt like the muscle was tired, the swallowing muscle was tired … some days it was better than others. (Patient 10)

One participant described more generally how acute mental illness had reduced his swallowing skills:

I'd gone really low you know in bipolar terms … … I couldn't … swallow at all … but it were physical as well as mental. (Patient 02)

This participant also reflected on his experiences of mealtimes on the ward. He reported that his illness had prevented his engagement in all aspects of eating including physical swallowing ability. An increased speed of eating was described by other participants who reported not being able to moderate their own pace:

I was filling my mouth faster than I could chew it and swallow it … like an automatic reflex … of shoving food in my mouth … and I kept eating at that pace but me mouth wouldn't [cope]. (Patient 07)

Participants reported that staff were generally unaware of swallowing difficulties and support was not offered.

## 5 Discussion

The results of this study highlight the heightened level of emotions associated with mealtimes in contrast with other ward activities and routines. The anxiety and stress experienced by participants directly influenced their pace and processes of eating and drinking, with a consequent impact on swallowing safety and incidence of choking. Currently there is scant attention in existing research relating to the assessment and management of psychosocial aspects of mealtimes generally, and particularly for adults with mental illness, outside of the more specific needs of those with eating disorders (Barnes et al. 2025). There is long‐standing attention to the mealtime support needs of people with dementia and older adults (Gibbs‐Ward and Keller 2005; Heikkilä et al. 2022; Li et al. 2021) but further research is indicated for stress management across populations (Kang et al. 2023). Supporting patients with psychosocial aspects of mealtimes falls within general good practices in nursing and wider care; raising awareness of the impact of mealtime stressors on nutrition and wider mental wellbeing should be integrated into recovery pathways (Blomberg et al. 2021; Nursing and Midwifery Council 2024; Pratt and Deeley 2025).

It is a concern that the results suggest that patients themselves are aware of the risk of choking but felt that staff lacked awareness relating to swallowing difficulties. Variation in the level of concern relating to choking has been highlighted in previous studies (Guthrie et al. 2015; Guthrie et al. 2023). Training in the supervision of mealtimes is available for staff in the UK with a focus on other patient groups and conditions (NHS England 2025). However, the findings of this study support the notion that staff teams on mental health wards and patients with mental illness are not routinely aware of dysphagia and that mealtime difficulties for adults with mental illness remain unrecognised (Guthrie and Stansfield 2015; Hemsley et al. 2019).

The results of this study also suggest that the loss of choices, cultural identity and personal customs associated with ward mealtimes is a further cause of distress. If mealtimes are viewed as a necessary task rather than a positive, comforting and individualised part of the day, mealtime enjoyment is reduced or eradicated. The patients' accounts of ward mealtimes reflected an impersonal, institutional, immutable system and suggested a sense of resignation and dependency. At home, meals may be arranged according to spiritual, religious or other family beliefs, customs and practices (Leslie and Lisiecka 2020). The important personal relationships and social aspects of mealtimes enjoyed prior to admission appear lost on admission to the wards, as mealtimes became routines based on staff norms and an institutional ‘blanket’ approach.

Participants' narratives highlighted that social and environmental aspects of mealtimes act as a catalyst on the ward, triggering feelings of frustration, stress and anxiety. Mealtimes were presented as negative influences on mental wellbeing: these emotions extended beyond the mealtime into their day‐to‐day mood and contributed adversely to their general recovery. In contrast, those who were able to prepare their own meals or take a more active role in helping at the meal table conveyed a sense of wellbeing, self‐esteem and achievement. This finding reflects studies exploring the role of meals with other patient groups (Gibbs‐Ward and Keller 2005; Leslie and Lisiecka 2020).

Studies relating to mealtimes in longer term stay in hospital or care home settings have primarily focussed on the importance of physical health benefits and nutritional intake (Naughton et al. 2021; Porter and Collins 2022). NHS guidance acknowledges this wider importance of food and drink in hospital settings (Shelley 2020; Pratt and Deeley 2025). In settings relating to older persons' care, studies have shown that with inclusive planning, mealtimes can become a shared positive experience for both patients and staff on the wards (Dickinson et al. 2008; Keller et al. 2015). To date, the wider benefits relating to psychosocial aspects of mealtimes have had scant attention in patients on mental health wards. Personal recovery models such as CHIME (Leamy et al. 2023) signpost the importance of connections, autonomy and empowerment to support recovery of mental wellbeing. However, the patient accounts reported in this study reflect a disconnect between clinical pathways aspiring to person‐centred mental health recovery and the stressful institutional practices on the wards at mealtimes.

Overall, staff attention to mental health recovery appeared to be suspended during mealtimes. Far from being a benign hiatus in rehabilitation, however, the results of this study suggest that mealtimes can form a damaging influence on patient mental wellbeing through escalating anxiety and other negative emotions. This is in stark contrast to home mealtimes, which offer comfort, soothing experiences and positive relationships. The benefits of understanding these aspects and supporting personalised mealtime approaches are relevant to current ward experiences as well as planning for recovery and future return to community living. This would be in keeping with the goals of rehabilitation, which should be

a whole systems approach to recovery from mental illness that maximises an individual's quality of life and social inclusion by encouraging their skills, promoting independence and autonomy in order to give them hope for the future and leading to successful community living. (Killaspy 2023, 51)

The results of our study indicate that the rehabilitative importance of personalised food and drink, whether shared with a group or taken in quieter surroundings, warrants greater recognition and integration into individual mental health recovery strategies.

## 6 Limitations and Directions for Future Research

This study supported participants from a marginalised population to voice their individual concerns (Serrant‐Green 2011). These insights are therefore presented for the reader to consider transferability and relevance in different settings and contexts (Thorne 2016). The findings of this study are limited by the relatively small number of participants, from only one mental health N.H.S. Trust, and should be interpreted with caution. The participants who volunteered were well enough to participate and results might not reflect the views of those too unwell to take part. Nonetheless, the experiences may be similar for other patients in the same situations. Supporting this marginalised population to reflect and comment using accessible resources and the communication expertise of a speech and language therapist researcher has offered insights into the unrecognised potential source of mental wellbeing that is mealtimes (Guthrie 2025). The relevance and importance of the findings reported here were confirmed by the service‐user advisory groups who supported this research. Further research is indicated to understand better the impact of stress and anxiety on the oral and pharyngeal stages of the swallowing process and to explore the impact of improving quality at mealtimes on patients' mental wellbeing. This is of particular relevance to adults with mental illness to understand how to integrate mental health recovery with mealtime processes and to develop individualised recovery strategies.

## 7 Conclusion

This study has explored patient perspectives on positive and negative aspects of mealtimes and how these influence mental wellbeing for adults with mental illness. Participants highlighted the heightened emotions prompted by negative aspects of mealtimes on the wards and consequent deterioration of mental well‐being. Patients' concerns with a lack of choice and loss of autonomy at mealtimes are not well recognised and are currently often overlooked in mental health recovery planning. Patients report their physical difficulties in swallowing are exacerbated by this heightened anxiety and stress. There is a missed opportunity to support recovery in patients by understanding how to individualise strategies for food and drink to promote both swallow safety and enhance mental health.

## 8 Relevance for Clinical Practice

Currently, medicalised perspectives influence the provision of mealtimes on mental health wards at the cost of more holistic approaches. There is a need to re‐focus attention onto more person‐centred and culturally aware support for mealtimes. Our findings should prompt ward staff in all roles to consider individual preferences and needs for food and drink. This should include environmental, social and cultural aspects with pace of eating carefully supported to promote safety and enjoyment. With individualised understanding and support, anxiety may be reduced, and consequently swallowing safety can be enhanced. The findings highlight the need for assessment and advice that integrate physical aspects with psychosocial concerns to inform and sustain wellbeing. Furthermore, personalised mealtimes can become an influential component of mental health recovery and inform strategies for mental and physical wellbeing which continue after discharge from hospital.

## Funding

The authors have nothing to report.

## Ethics Statement

Ethical approval was provided by the Health Research Authority: REC reference 21/YH/0038 IRAS project ID 270116.

## Conflicts of Interest

The authors declare no conflicts of interest.

## Supporting information


**Appendix S1:** inm70212‐sup‐0001‐AppendixS1.docx.

## Data Availability

The data that support the findings of this study are available on request from the corresponding author. The data are not publicly available due to privacy or ethical restrictions.
