# Supplementary material for: Patient Perspectives on Inpatient Mealtimes: Insights on Swallowing, Mental Wellbeing and Recovery
Source: Int J Ment Health Nurs. 2026 Jan 5;35(1):e70212. doi: 10.1111/inm.70212 (PMC12766665; doi:10.1111/inm.70212)
Supplement: Supplementary file 1 — Appendix S1: inm70212‐sup‐0001‐AppendixS1.docx. [file INM-35-0-s001.docx]

# Appendix

Consolidated criteria for reporting qualitative studies (COREQ): 32-item checklist (Tong, Sainsbury et al. 2007)

Domain 1: Research team and reflexivity. Personal Characteristics

| **COREQ item** | Responses for this study | **Additional comments from Braun & Clarke’s checklist and guidance** |
| --- | --- | --- |
| **1. Interviewer/facilitator. Which author/s conducted the interview or focus group?** | Researcher SG conducted all interviews (in person or online options offered). | **Does the researcher ‘’own their perspectives’’?**  Reflexivity throughout |
| **2. Credentials. What were the researcher’s credentials? E.g. PhD, MD** | PhD student, supported by supervisory team (University of …. ) |  |
| **3. Occupation. What was their occupation at the time of the study?** | Clinical academic. Presence on wards due to clinical role but no participants on active caseload. |  |
| **4. Gender. Was the researcher male or female?** | Female, acknowledged in text |  |
| **5. Experience and training. What experience or training did the researcher have? Relationship with participants** | 35 years clinical experience  Research training through university and support from doctoral supervisors. Participants varied in terms of familiarity with SG, patients were supported by ward staff to understand nature and details of study.  Researcher reiterated nature of role and study |  |
| **6. Relationship established.**  **Was a relationship established prior to study commencement?** | Support for participants offered before interview commenced, ongoing support during interview.  No patients on active caseload were interviewed. |  |
| **7. What did the participants know about the researcher? e.g. personal goals, reasons for doing the research.** | Participant info sheet offered and explained.  Further support to understand study during interview.  Accessible information |  |
| **8. Interviewer characteristics. What characteristics were reported? e.g. Bias, assumptions, reasons, interests in the research topic.** | Reflexivity completed, journal supporting reflection after interviews and during analysis |  |

Domain 2: study design

| **Theoretical framework** |  | **Braun & Clarke guidance** |
| --- | --- | --- |
| **9. Methodological orientation and Theory.**  **What methodological orientation was stated to underpin the study? e.g. grounded theory, discourse analysis, ethnography, phenomenology, content analysis.** | Qualitative exploratory study.  Pragmatic approach, interpretive description  Reflexive thematic analysis (RTA) (Braun and Clarke 2022) | **Consistency between research aims, theory and use of RTA?**  Reviewed in supervision  Explicit description in methodology chapter,  RTA approach described in table (method chapter) |
| **Participant selection**  **10. Sampling. How were participants selected? e.g. purposive, convenience, consecutive, snowball.** | Convenience sample - Patients across (city) |  |
| **11. Method of approach. How were participants approached? e.g. face-to-face, telephone, mail, email.** | Patients and staff – ward posters and staff email, in person discussion at meetings  . | **Clear explanation of use of RTA? Good fit between data collection and RTA use?**  Full details in methods section. |
| **12. Sample size. How many participants were in the study?** | 13 total |  |
| **13. Non-participation. How many people refused to participate or dropped out? Reasons? Setting?** | Refusals not known  All interviews completed, 2 patients asked to finish early. |  |
| **14. Setting of data collection. Where was the data collected? e.g. home, clinic, workplace.** | Inpatient wards across (city) |  |
| **15. Presence of non-participants. Was anyone else present besides the participants and researchers?** | Patients were offered staff support but none required |  |
| **16. Description of sample. What are the important characteristics of the sample? e.g. demographic data, date of Data collection?** | Demographics collected at time of interview - anonymised |  |
| **17. Interview guide. Were questions, prompts, guides provided by the authors? Was it pilot tested?** | Semi structured interview derived from suggestions by service-user group, also from clinical experience.  Pilot testing – first 3 interviews discussed with supervisors. |  |
| **18. Repeat interviews. Were repeat interviews carried out? If yes, how many?** | Repeat not feasible due to fluctuations in wellbeing, Covid restrictions, transient population. |  |
| **19. Audio/visual recording. Did the research use audio or visual recording to collect the data?** | Audio recording of interviews, MSTeams transcription for online interviews.  Transcription completed and checked by SG with oversight from supervisors. |  |
| **20. Field notes. Were field notes made during and/or after the interview or focus group?** | Notes made during and after interviews, also during transcription. |  |
| **21. Duration.**  **What was the duration of the interviews or focus group?** | Duration (minutes) ranged from 6:04 - 41:58 |  |
| **22. Data saturation.**  **Was data saturation discussed?** | Sample size was feasible and sufficient given timescale and allowed in depth analysis.  Saturation not compatible with qualitative approach – this study completed in depth analysis and reflexive transparency allowing reader to consider transferability. |  |
| **23. Transcripts returned. Were transcripts returned to participants for comment and/or correction?** | Not feasible. Comments invited from user group on relevance and confirmability |  |

Domain 3: analysis and findings

| **Data analysis 24. Number of data coders. How many data coders coded the data?** | RTA used – SG coded and presented draft codes to supervisors for discussion |  |
| --- | --- | --- |
| **25. Description of the coding tree. Did authors provide a description of the coding tree?** | Nvivo used to list, cross reference and explore codes. | **Clear outline of themes as map or table**  yes |
| **26. Derivation of themes.**  **Were themes identified in advance or derived from the data?** | Inductive analysis deriving themes from data. Iterative process returning to audio and transcripts to ensure close match between data and themes. | **RTA process consistent**  **Themes not ‘domain summaries’, sufficiently indepth analysis**  Iterative analysis moved to latent themes in discussion with supervisors |
| **27. Software: What software, if applicable, was used to manage the data?** | Nvivo |  |
| **28. Participant checking.**  **Did participants provide feedback on the findings? Reporting of this?** | Themes presented to user groups for discussion and comment. |  |
| **29. Quotations presented.**  **Were participant quotations presented to illustrate the themes / findings? Was each quotation identified? e.g. participant number.** | Participant reference codes presented identifying interview group and number. |  |
| **30. Data and findings consistent.**  **Was there consistency between the data presented and the findings?** | Coding and thematic analysis presented to supervising team for reflection and feedback. Also presented to service user groups for discussion of credibility. | **Do the reported themes give rise to actionable outcomes? Is this consistent with qualitative approach?**  Transferability discussed in discussion, also implications for future research and clinical practice. |
| **31. Clarity of major themes. Were major themes clearly presented in the findings?** | Themes presented as network (figure 1) |  |
| **32. Clarity of minor themes. Is there a description of diverse cases or discussion of minor themes?** | Presented as figure 1 showing themes |  |

Braun, V. and V. Clarke (2022). Thematic analysis: a practical guide, Sage

Tong, A., P. Sainsbury and J. Craig (2007). "Consolidated criteria for reporting qualitative research (COREQ): a 32-item checklist for interviews and focus groups." International Journal for Quality in Health Care **19**(6): 349-357.
